# Safety of the Radiofrequency Balloon for Pulmonary Vein Isolation: A Focus on Lesion Metric Analysis of Posterior Electrodes

**DOI:** 10.3390/jcm12196256

**Published:** 2023-09-28

**Authors:** Alexandre Almorad, Alvise Del Monte, Yannick Teumer, Milad El Haddad, Luigi Pannone, Domenico Giovanni Della Rocca, Charles Audiat, María Cespón-Fernández, Sahar Mouram, Robbert Ramak, Ingrid Overeinder, Gezim Bala, Antonio Sorgente, Erwin Ströker, Juan Sieira, Pedro Brugada, Mark La Meir, Carlo de Asmundis, Gian-Battista Chierchia

**Affiliations:** 1 Heart Rhythm Management Centre, Postgraduate Program in Cardiac Electrophysiology and Pacing, European Reference Networks Guard-Heart, Universitair Ziekenhuis Brussel-Vrije Universiteit Brussel, 1090 Brussels, Belgium; alvise.delmonte@gmail.com (A.D.M.); lui.pannone@gmail.com (L.P.); domenicodellarocca@hotmail.it (D.G.D.R.); charles.audiat@gmail.com (C.A.); maria.cespon.fernandez@gmail.com (M.C.-F.); mouram.sahar@gmail.com (S.M.); robbert.ramak@uzbrussel.be (R.R.); ingrid.overeinder@uzbrussel.be (I.O.); gezim.bala@uzbrussel.be (G.B.); sorgente.antonio@gmail.com (A.S.); erwin.stroker@uzbrussel.be (E.S.); juan.sieira@uzbrussel.be (J.S.); pedro@brugada.org (P.B.); jeanbaptiste.chierchia@uzbrussel.be (G.-B.C.); 2 Department of Medicine II, Ulm University Medical Center, 89070 Ulm, Germany; yannick.teumer@uniklinik-ulm.de; 3 Independent Researcher, 00790 Helsinki, Finland; milad.elhaddad@emdt.eu; 4 Cardiac Surgery Department, Universitair Ziekenhuis Brussel-Vrije Universiteit Brussel, 1090 Brussels, Belgium; mark.lameir@uzbrussel.be

**Keywords:** pulmonary vein isolation, radiofrequency balloon, posterior wall, esophageal temperature rise

## Abstract

Previous clinical studies on pulmonary vein isolation (PVI) with radiofrequency balloons (RFB) reported safe and effective procedures for a 20 s RF delivery via posterior electrodes. Recent recommendations from the manufacturer suggest reducing the application time to 15 s on the posterior wall (PW) when facing the esophagus region. Here, we retrospectively assess whether 15 s of RF delivery time on posterior electrodes is safe while still ensuring lesion metrics of sufficient quality. This retrospective study included 133 patients with paroxysmal and persistent atrial fibrillation who underwent PVI using an RFB (Heliostar, Biosense Webster, Inc., Irvine, CA, USA) at two European centers. The ablation protocol was set for an RF duration of 20 s/60 s for the posterior/anterior electrodes. A multielectrode temperature probe was systematically used. In the case of an esophageal temperature rise (ETR) above 42 °C (ETR+), an endoscopic evaluation was performed. All posterior electrode lesion metric dynamics (temperature (T) and impedance (Z)) were collected from the RFB generator and analyzed offline. In total, 2435 posterior electrode applications were analyzed. With an RF delivery of 19.8 (19.7–19.8) s, the median impedance drop was 18.4 (12.2–25.2) Ω, while the temperature rise was 11.1 (7.1–14.9) °C. Accordingly, impedance (84.6 (79.3–90.2) Ω) and temperature plateaus (38 (35.3–41.1) °C) were reached at 13.9 (10.6–16) s and 16.4 (12.6–18.5) s, respectively. Overall, 99.6% and 95.8% of electrodes reached 90% (16.6 Ω) and 95% (17.5 Ω) of their impedance drops within 15 s of RF delivery, while 97.2% and 92.8% achieved 90% (34.2 °C) and 95% (36.1 °C) of their temperature rise to reach the plateaus within 15 s of RF delivery. An ETR >42 °C occurred in 37 (30.1%) patients after 17.7 ± 2.3 s of RF delivery. In the ETR+ group, the impedance drop and temperature rise on the posterior electrodes were higher compared to patients where ETR was <42 °C. Two asymptomatic thermal esophageal injuries were observed. In conclusion, 15 s of RF delivery on the posterior electrodes provides a good balance between safety, with no esophageal temperature rise, and efficacy with high-profile lesion metrics.

## 1. Introduction

Atrial fibrillation (AF) is the most common atrial arrhythmia, affecting up to 2% of the population, and its prevalence has increased three-fold over the last 50 years [1]. Pulmonary vein isolation (PVI) has proven to be an effective treatment option for paroxysmal AF [2,3]. The legacy ablation technique adopted to deliver energy all around the ostium of pulmonary veins (PVs) is the “point-by-point” technique, via a focal catheter guided by a 3D mapping system. To address the increasing demand for ablation treatment in AF patients, new alternative technologies allowing a shorter learning curve and faster procedures have been made available. For instance, cryoballoon (CB) ablation (CBA) has become one of the standard techniques in single-shot devices for PVI. It has been demonstrated to be both safe and effective while yielding improved procedural consistency and outcomes, leading to its widespread adoption [4,5,6]. 

Recently, a new single-shot catheter, namely the radiofrequency balloon (RFB, Heliostar, Biosense Webster, CA, USA), has been brought to market. Unlike CBs, RFBs are based on thermal energy via radiofrequency (RF) and are fully integrated with an electroanatomic mapping system (CARTO^®^3, Biosense Webster, CA, USA). Moreover, RFBs allow for a more customized energy delivery as the operator can independently activate all or only some of the electrodes to titrate RF to specific anatomical regions and avoid overshooting where not required [7,8]. 

However, the main safety concern with thermal energy use is overheating of the esophagus when ablating the posterior wall (PW) of the left atrium (LA) due to excessive amounts of RF energy being transferred to the PW. Specifically, with the RFB, the energy transfer to the tissue depends on several parameters; some can be controlled by the operator, such as balloon positioning and RF delivery time, while others cannot, like power preset, force applied to the tissue, and baseline impedance. 

Previous clinical studies on PVI with the RFB [8,9,10,11] reported effective and safe procedures with RF delivery of 15 W unipolar for 20 s on the PW and for 60 s through the other electrodes. Recent recommendations from the manufacturer, however, suggest reducing the application time to 15 s on the PW when facing the esophagus region. 

This retrospective study aims to assess whether a 15 s RF delivery time on the posterior electrodes is safe while still ensuring lesion metrics of sufficient quality.

## 2. Materials and Methods

### 2.1. Study Population

In this retrospective, multicenter study, all 133 patients with paroxysmal and persistent AF scheduled for PVI using the RFB (Heliostar, Biosense Webster, Inc., Irvine, CA, USA) in two European centers between May and November 2022 were included in the analysis. 

The study was conducted in accordance with the ethical principles established by the Declaration of Helsinki in its revised 2013 version, and was approved by the ethics committees of the two participating institutions.

### 2.2. PVI with the RFB

Below, we briefly outline PVI performed with the RFB as previously described [7]. Patients were treated under general anesthesia or deep sedation and underwent uninterrupted anticoagulation therapy. Femoral access was performed under echo guidance. An esophageal multisensor temperature probe (CIRCA multisensor, Abbott, Chicago, IL, USA) was used in every patient to monitor the temperature increase in the esophagus. 

A diagnostic catheter was introduced inside the coronary sinus to monitor atrial electrograms and allow pacing from the superior vena cava (SVC) during right pulmonary vein ablation. Then, a single transseptal puncture was performed under transesophageal echography or fluoroscopic guidance using a “fixed sheath, immediately followed by bolus heparin administration to maintain the activated clotting time in the range of 300–350 s throughout the entire procedure.

A pre-ablation electroanatomical map of the PVs and PW was created using a flexible circular mapping catheter (Lasso NAV, Biosense Webster, CA, USA) through a fixed sheath; this was then exchanged for a deflectable sheath (GuideStar, Biosense Webster, CA, USA) and a balloon with a dedicated circular catheter (LassoStar, Biosense Webster, CA, USA).

During the study period, this circular catheter was updated with mapping capacities (LassoStar™ NAV; Biosense Webster, CA, USA), which made it usable through the RFB and allowed us to avoid using an extra catheter. The mapping was performed directly after the sheath exchange.

**Figure 1:**
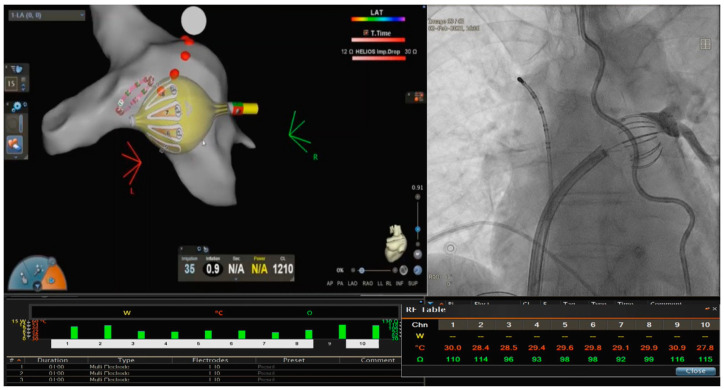
Example of RFB positioning. Example of optimal balloon positioning in LIPV: inflation index > 0.8; baseline impedance across 10 electrodes ranging between 80 and 110 Ω; and baseline temperature < 31 °C in all electrodes. Electrodes #6, #8, and #9 are selected as posterior electrodes.

Once the map had been created, a circular catheter (LassoStar or LassoStar NAV) was anchored inside the PV to record its potentials. Subsequently, the balloon was optimally positioned for ablation coaxially to the PVs and electrode–tissue contact was assessed using either fluoroscopy and dye injection or the following pre-ablation parameters [7,11]:

- Inflation index > 08
- Baseline impedance of the 10 electrodes, ranging between 90 and 120 Ω with a variability of ≤20 Ω across electrodes (Figure 1);
- Electrode temperature <31 °C with a variability of ≤3 °C across electrodes (Figure 1).

Once optimally positioned, RF energy was delivered simultaneously in unipolar mode to the 10 electrodes using an nGEN generator (Biosense Webster Inc., Israel) equipped with two amplifiers and 10 different RF channels. The power was set to 15 W in the temperature control mode with a target temperature of 55 °C, as monitored by the Temperature Control Loop. The irrigation flow rate was set to 35 mL/min during RF energy delivery (5 mL/min when RF was off). During ablation, PV potentials were monitored using a circular diagnostic catheter to evaluate real-time isolation.

Based on the 3D electroanatomical map, posterior electrodes were identified and selected directly on the generator. All remaining electrodes were considered anterior. RF energy delivery duration depended on the anatomical region: 20 s through the posterior and 60 s via the anterior electrodes. In the present study, two to three electrodes were usually set as posterior at the discretion of the treating physician and based on the patient’s anatomy. 

The metrics of all lesions, including the duration of RF delivery, impedance, and temperature for each posterior electrode, were collected from the RFB generator and analyzed offline.

### 2.3. RFB Architecture and Lesion Metrics

**Figure 2:**
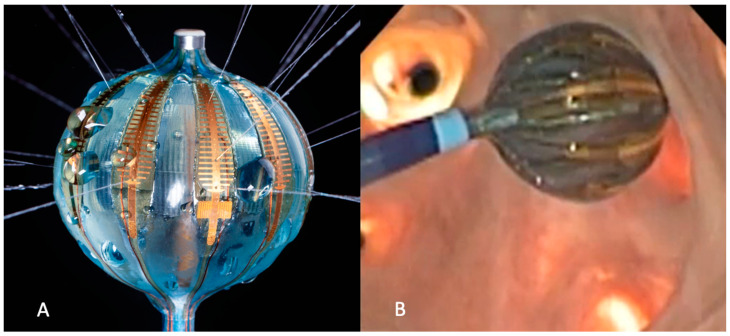
RFB electrodes and contact assessment. (**A**) Heliostar balloon fully inflated and irrigated. (**B**) Image from inside a heart model. Impedance/temperature values vary according to the electrode’s surface contact. High impedance/low temperature may reflect enhanced contact, while low impedance/high temperature may indicate that the electrode is in the blood pool.

The RFB used in all procedures was a 28 mm compliant balloon, equipped with a central lumen for irrigation and inflation and 10 flexible gold-plated electrodes, each with four irrigation holes and a thermocouple positioned in the middle of the longitudinal axis of the electrode (Figure 2A). Electrode temperature (T) represents the temperature measured over the entire tissue–electrode interface.

The impedance (Z) depends on the surface of the electrode in contact with the tissue (Figure 2B) and on the position of the indifferent electrodes [12], as well as the tissue thickness. 

Raw data, including power delivery, temperature, and impedance, were stored in the generator. Temperature (T(t)) and impedance (Z(t)) variations over time were extracted from each electrode and ablation session for offline analyses. 

**Figure 3:**
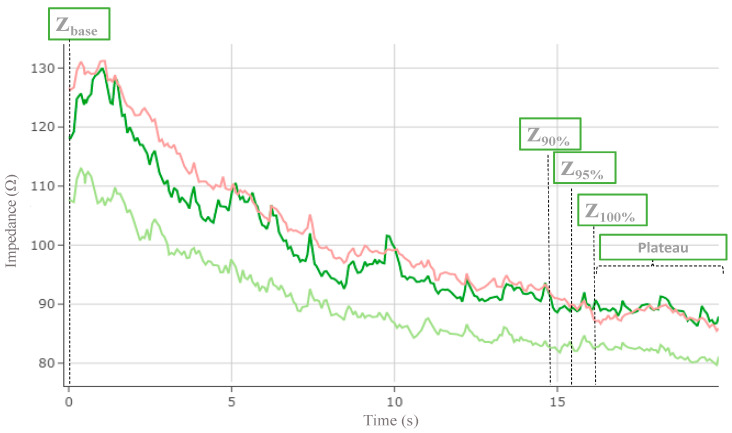
Example of impedance drop on 3 posterior electrodes during a single 20 seconds of radiofrequency delivery. Impedance progression over 20 seconds of RF delivery time represented from the baseline (Z_base_) to each percentile of the plateau (Z_90%_, Z_95%_, Z_100%_).

**Figure 4:**
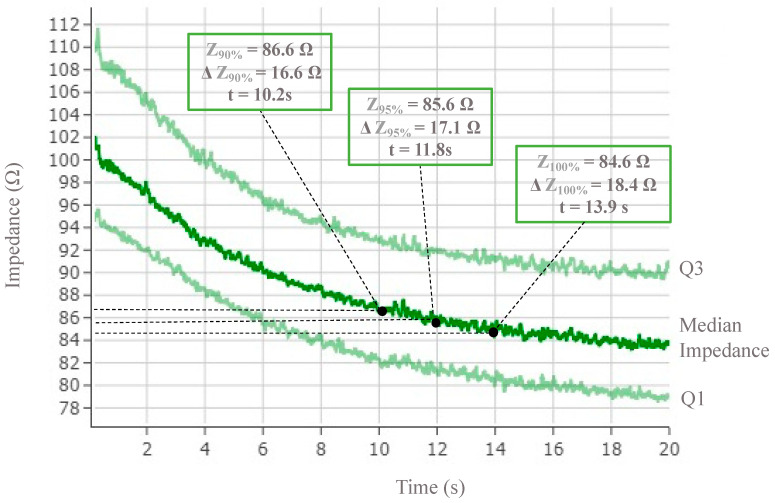
Impedance drop over time for all posterior radiofrequency electrodes. Median (Q1–Q3) impedance progression on all posterior electrodes over 20 s of RF delivery time. Impedance absolute values and drop are marked at each percentile of the plateau (90%, 95%, 100%). The time to reach these metric values is also represented.

**Figure 5:**
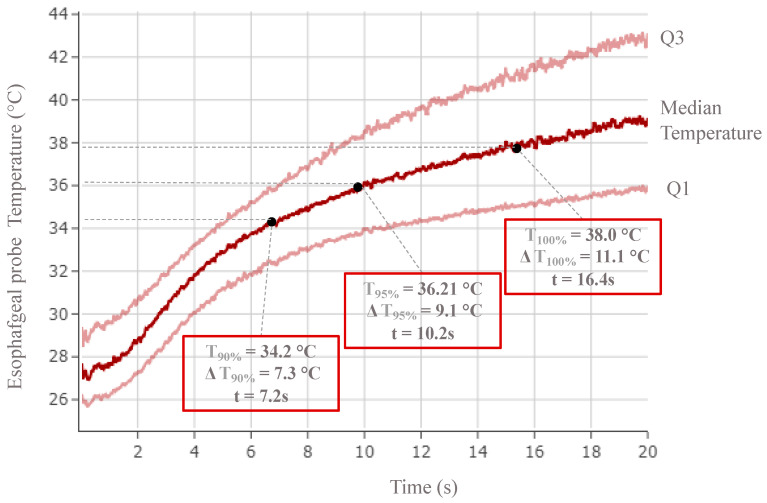
Temperature rise over time for all posterior radiofrequency electrodes. Median (Q1–Q3) temperature progression on all posterior electrodes over 20 s of RF delivery time. Temperature absolute values and drop are marked at each percentile of the plateau (90%, 95%, 100%). The time to reach these metric values is also represented.

We defined the plateau of temperature T(t) = T_100%_ or impedance Z(t) = Z_100%_ where T_100%_ and Z_100%_ are calculated based on the 95% quantile of the temperature or impedance values throughout the ablation session. This plateau correspond to the first instance in time when these values show any significant temporal variations until the end of the ablation session. Alternatively, a plateau of temperature (T_100%_) or impedance (Z_100%_) can be defined as the period of time between the function reaching its maximum until the end of the RF session (Figure 3, Figure 4 and Figure 5).

### 2.4. Esophageal Temperature Monitoring (ETM)

Both centers participating in this study used the same esophageal multisensor temperature probe (CIRCA multisensory, Abbott) with the esophageal temperature (ET) alarm set to 39 °C. Baseline ET and ET_max_ values were systematically recorded. All patients with ETR > 42 °C were referred for endoscopy within 10 days of the index procedure.

### 2.5. Analytics and Statistics

We collected the following parameters for each posterior electrode: baseline impedance (Z_base_) and temperature (T_base_), impedance at the plateau (Z_100%_) and its 90th (Z_90%_) and 95th (Z_95%_) percentile values, and temperature at the plateau (T_100%_) and its 90th (T_90%_) and 95th (T_95%_) percentile values. The impedance drop at the plateau (ΔZ_100%_) and temperature rise to reach the plateau (ΔT_100_) were also computed with their 90th (ΔZ_90%_; ΔT_90%_) and 95th (ΔZ_95%_; ΔT_95%_) percentiles. The time required to reach each value was calculated for every posterior electrode. All values were reported as medians (Q1–Q3). 

Patients with ETR > 42 °C were included in the ETR+ group, whereas those with ETR < 42 °C were included in the ETR- group.

The following metrics were compared between the two groups (ETR+/ETR-) to determine whether there were any statistically significant differences:

- Baseline impedance (Z_base_) and temperature (T_base_),
- Times to reach each impedance drop (ΔZ_90%_, ΔZ_95%_, ΔZ_100%_) and temperature rise (ΔT_90%_, ΔT_95%_, ΔT_100%_) percentiles,
- Absolute impedance at 15 s, (Z(15)_90%_, Z(15)_95%_, Z(15)_100%_) and temperature (T(15)_90%_, T(15)_95%_, T(15)_100%_) values,
- Impedance drop at 15 s, (ΔZ(15)_90%_, ΔZ(15)_95%_, ΔZ(15)_100%_) and temperature rise (ΔT(15)_90%_, ΔT(15)_95%_, ΔT(15)_100%_),
- Impedance drop at 20 s (ΔZ(20)_90%_, ΔZ(20)_95%_, ΔZ(20)_100%_) and temperature rise (ΔT(20)_90%_, ΔT(20)_95%_, ΔT(20)_100%_).

For ETR+, temperature rise and impedance drop at 15 and 20 s (ΔZ(15)_100%_ vs. ΔZ(20)_100%_) and ΔT(15)_100%_ vs. ΔT(20)_100%_) were compared.

For each group, a Shapiro–Wilk normality test was performed. A Kruskal–Wallis (non-parametric) test was used to evaluate differences between groups. A *p*-value < 0.05 was used to identify statistically significant difference between both groups.

## 3. Results

### 3.1. Lesion Metrics

A total of 133 cases were used for lesion metric analysis, involving 2435 posterior electrodes. The baseline impedance was 102.4 (95.8–111.1) Ω, while the baseline temperature was 27.1 (25.8–28.9) °C. Overall, RF delivery lasted 19.8 (19.7–19.8) s with an impedance drop of 18.4 (12.2–25.2) Ω and a temperature rise of 11.1 (7.1–14.9) °C. Accordingly, an impedance plateau of 84.6 (79.3–90.2) Ω was reached after 13.9 (10.6–16) s and a temperature plateau of 38 (35.3–41.1) °C after 16.4 (12.6–18.5) s (Table 1).

It took 10.2 (7.9–11.9) and 11.8 (9.1–13.8) s to reach ΔZ_90%_ (16.6 (11–22.7) Ω) and ΔZ_95%_ (17.5 (11.6–24) Ω), respectively (Table 1). The corresponding times for ΔT_90%_ (7.3 (3.7–19.8) °C) and ΔT_95%_ (9.1 (5.3–12.8) °C) were 7.2 (3.9–9.6) and 10.1 (6.4–13.1) s, respectively (Table 1).

Of note, 99.6% and 95.8% of the posterior electrodes reached 90% (16.6 Ω) and 95% (17.5 Ω) of their impedance drops within 15 s of RF delivery (Table 1), while 97.2% and 92.8% achieved 90% (34.2 °C) and 95% (36.1 °C) of their temperature increase within 15 s of RF delivery (Table 1). 

### 3.2. Lesion Metrics in Posterior Electrodes vs. Esophageal Temperature Monitoring Analysis

Esophageal temperature rise (ETR+ > 42°C) occurred in 37 (30.1%) patients after 17.7 ± 2.3 s of RF delivery, whereas an ETR ≥ 39 °C was seen in 52 (64.5%) patients after 13.2 ± 3.3 s. ETR was mainly (28, 76.3%) observed around the inferior PV (14 left vs. 14 right) and less frequently (9, 23.7%) around the superior PV (6 left and 3 right). 

Following the internal protocol, all patients in the ETR+ group underwent gastroscopy after an average of 7 ± 4 days. In two cases, procedure-related esophageal erythema was observed and was uneventfully treated with drug therapy.

Compared to ETR-, the 78 ETR+ electrodes exhibited a lower baseline temperature of 26.1 °C (25.1–28.1) vs. 27.3 °C (26–29) (*p* < 0.0001) and a higher baseline impedance of 105.1 (98.3–115.5) Ω vs. 101.8 (95.5–110.5) Ω (*p* = 0.018) (Table 2).

In the ETR+ group, a lower impedance Z_100%_ (82.2 (77.1–87.5) Ω vs. 84.8 (79.4–90.6) Ω, *p* = 0.012) was reached after a longer time (14.7 (12.3–16.4) s vs. 13.6 (10–15,9) s, *p* = 0.005) when compared with ETR– group (Table 2).

In the ETR+ group at T_100%_, higher temperatures (*p* = 0.002) were reached at similar times (*p* = 0.47) compared with ETR−: 13.6 (10–15.9) s to reach 39.0 (36.3–42.9) °C vs. 16.3 (12.3–18.4) s to reach 38.0 (35.3–41.3) °C (Table 2).

However, at T_90%_ and T_95%_, higher temperatures (*p* = 0.001 and *p* = 0.002) were reached later (*p* = 0.0007 and *p* = 0.004) in the ETR+ group vs. ETR− (8.6 and 11.3 s to reach 35.1 °C and 37 °C vs. 7.1 and 10 s to reach 34.2 °C and 36.1 °C, respectively) (Table 2).

At t = 15 s, the absolute temperature (T(15)) (*p* = 0.034), temperature rise (ΔT(15)) (*p* = 0.003), and impedance drop (ΔZ(15)) (*p* < 0.0001 ) were all higher in the ETR+ group, whereas absolute impedance (Z(15)) was lower (*p* < 0.001), regardless of the plateau percentiles (Table 3).

In the ETR+ group, all (100%) electrodes reached 90% of their temperature/impedance plateaus before 15 s (T (15)_90%_, Z (15)_90%_), whereas only 46.1%/71.8% reached 100% of their temperature/impedance plateaus before 15 s (T (15)_100%_, Z (15)_100%_).

No statistically significant differences were observed in the ETR+ group between 15 and 20 s for either the impedance drop (ΔZ (15) vs. ΔZ (20), *p* = 0.31) or the temperature rise (*p* = 0.11) (Table 4).

## 4. Discussion

The current study is the first to address the safety of the esophagus during PVI performed with RFB by analyzing posterior electrode metrics and ETR during RF delivery. The major findings are that: (1) ETR occurs after an average duration of 17.7 ± 2.3 s of RF delivery; (2) impedance drop and temperature rise are higher when ETR > 42 °C occurs; (3) 15 s of RF delivery to the posterior electrodes provides a good balance between safety (no ETR) and sufficient lesion metrics; and (4) minimal procedure-related esophageal lesions were observed.

### 4.1. PVI and Esophageal Injury in Context

Esophageal injuries are the Achilles’ heel of RF catheters ablations, as they can produce transmural lesions. Esophageal ulceration, considered a surrogate marker of the risk of clinically relevant esophageal complications, such as perforation and fistula, is as high as 9.3% after catheter ablation [13]. While Wolf et al., reported a low rate of esophageal injury (1.2%) using 35 W at the PW in close-guided ablation [14], other procedures more frequently produced asymptomatic esophageal erythema/ulcers, namely AF ablation with focal tip RF catheters (in 5 to 40% of patients) [2], CB ablation (in 3.2 to 18.8% of patients) [15] and, more recently, RFB ablation (in 7.7–12.8% of patients) [8,10,11,16,17]. Recently, 3D reconstruction of the esophagus using the mean of intracardiac echocardiography during AF ablation was shown to be safe and feasible [18].

Regardless of the technology used, atrio-esophageal fistulas have been observed in 0.02–0.11% of patients [2]. A recent registry-based study [19] reported an average atrio-esophageal fistula rate of 0.025% (0.038% with RF, 0.0015% with cryo, and 0.025% with other approaches). 

To avoid the risk of esophageal injury, the total amount of RF energy delivered to the PW, and thus to the esophagus, can be reduced by decreasing RF power and/or RF delivery time and/or force [2]. 

Recently, a novel RFB catheter with the ability to customize energy delivery for each anatomical region was introduced to the market. By selectively reducing RF delivery time on all or some electrodes, thermal energy delivery is titrated, thus avoiding overshooting on fragile anatomical segments, such as the PW. 

To the best of our knowledge, this is the first study to investigate the dynamics of posterior electrodes’ RF lesion metrics based on impedance and electrode temperature evolution during RF delivery. The aim of this study was to assess whether the amount of energy delivered to the PW can be decreased, thus sparing the esophagus, without compromising acute efficacy.

### 4.2. Optimal RF Delivery Time for Best Lesion Metrics

For an RF delivery of 20 s through posterior electrodes, RFB lesion metrics showed the impedance reaching a plateau of 84.8 (79.3–90.3) Ω after 13.9 (10.6–16) s, while temperature plateaued at 38 (35.3–41.4) °C after 16.4 (12.6–18.46) s (Table 1 and Table 2). Once impedance had plateaued, it dropped by 18.4 (12.2–25.2) Ω, while temperature increased by 11.1 (7.1–14.9) °C (Table 1).

Previous reports [8,9,14] recommended post-ablation impedance drops of >12 Ω and temperature increases of >6 °C. In a recent publication by Del Monte et al. [8], an impedance drop of >19.2 Ω and a temperature rise of >11.1°C were possible predictors of acute persistent single-shot isolation, although in the latter case, no separate analysis was performed for posterior and non-posterior electrodes. Given the thinner tissue characterizing the LA–PV junction posteriorly compared to other anatomical segments (roof, anterior ridge, and inferior) [20,21], it would appear reasonable to aim for a lower impedance drop and temperature rise to preserve adjacent structures while still achieving durable lesions [22,23]. Therefore, we computed the lesion metrics of posterior electrodes throughout RF delivery by assessing the 90th, 95th, and 100th percentiles of impedance, impedance drop, temperature, and temperature rise plateaus.

We found that 90% (16.6 Ω) and 95% (17.5 Ω) impedance drops were reached relatively quickly after 10.2 and 11.8 s, respectively (Table 1). On the other hand, temperature achieved 90% (7.3 °C) and 95% (9.1 °C) of its increase to reach the plateau after just 7.2 and 10.1 s, respectively (Table 1).

In other words, a relatively short RF application of approximately 12 s would suffice to guarantee sufficient lesions. 

Our lesion metric analysis of RF delivery showed that 99.6% and 95.8% of electrodes achieved 90% (16.6 Ω) and 95% (17.5 Ω) of their impedance drops from the plateau before 15 s, respectively (Table 1). Similarly, at t < 15 s, 97.2% and 92.8 % of the electrodes achieved 90% (7.3 °C) and 95% (9.1 °C) of their temperature rise plateau, respectively (Table 1). Consequently, increasing RF duration from 15 to 20 s would only marginally improve the lesion profile. 

Interestingly, after 15 s, 75.6 % of electrodes had reached the full impedance drop plateau, while only 47.0% had reached their temperature plateau. This emphasizes that, at 15 s, most lesions had already reached their maximum efficacy, and an additional 5 s of RF delivery could be considered overshooting. Recently, the RFB manufacturer (Biosense Webster) instructed operators to apply RF on the posterior electrodes for only 15 s instead of 20 s in cases where the posterior electrodes are facing the esophagus.

Based on these observations, where the large majority of posterior electrodes achieved ‘excellent’ lesion metrics (impedance drop > 12 Ω, temperature rise > 6 °C), it is reasonable to consider 15 s as the new target RF delivery time for posterior electrodes, potentially without compromising efficacy.

### 4.3. Optimal Indicators to Spare the Esophagus Temperature rise

While data on RFB usage remain scarce, the most recent studies on the subject did not report any esophageal lesions [7,8,9,14]. With ETR >42 °C being reached after 17.7 ± 2.3 s of RF delivery, we analyzed the behavior of lesion metrics/characteristics (impedance drop and temperature rise) to prevent such high ETR and thus esophageal injury. 

Comparing patients with and without ETR (ETR+ vs. ETR-), ETR+ patients exhibited a lower electrode baseline temperature (26.1 vs. 27.3 °C, *p* <0.001) and higher baseline impedance (105.1 vs. 101.8 Ω, *p* = 0.018) (Table 2). While this could be due to tissue characteristics, it seems more likely that the initial position of the RFB also plays an important role. A lower baseline electrode temperature and higher baseline impedance may indicate a better contact with the tissue, likely due to a higher pressure on the balloon before applying RF. 

Moreover, even for RF delivery times of 15 s, both the impedance drop and temperature rise were significantly higher in the ETR+ group (*p* < 0.001 and *p* < 0.002, respectively; Table 3), suggesting different lesion growths in both groups. Several factors might influence these dynamics, such as the initial positioning of the RFB and the proximity of another anatomical structure such as the esophagus, which could impede the cooldown process of the PW and in turn lead to a greater temperature increase at the tissue–electrode interface during RF delivery. Furthermore, in both groups, most (≥92%; Table 3) electrodes reached their 95% impedance drop/temperature rise of their respective plateaus before 15 s, confirming our conclusion that lesion growth is already achieved before 15 s. 

Considering these findings, and based on our proposal to reduce the total amount of energy delivered to the PW to avoid ETR >42 °C, which occurred at 17.7 ± 2.3 s, we assessed whether RF delivery time could be reduced to 15 s. Specifically, we found that the impedance drop at t = 15 s was consistently higher in the ETR+ (21.4, 22.2, and 22.4 Ω) than in the ETR- group (16, 16.6, and 15.4 Ω), regardless of the percentile (90%, 95%, and 100% impedance drop), suggesting very advanced lesion growth before 15 s (Table 3). In addition, as lesion metrics (e.g., impedance drop and temperature rise) are nearly identical at 15 and 20 s in the ETR+ group (Table 4), delivering RF for an additional 5 s does not seem to result in any measurable benefit in terms of efficacy, suggesting that RF delivery for more than 15 s may lead to overshooting.

Accordingly, an RF delivery duration of 15 s could be considered a valid option for posterior electrodes, as all safety and efficiency lesion metric criteria are fulfilled.

## 5. Limitations

This study has several limitations. (1) This study was limited to posterior electrodes’ lesion metrics, and so no conclusion on the entire RFB electrodes can be drawn; (2) this was a retrospective study with offline data analysis, and the suggested values must be validated prospectively; (3) the number of posterior electrodes varied between two and three, following the procedures; and (4) ETR ≥ 42°C has been considered an acceptable cut-off to request a follow-up endoscopy. Other analyses may be needed to validate these observations with lower cut-off values. The number of patients who underwent endoscopy was limited, and further studies are needed to assess the safety of RFB.

## 6. Conclusions

This retrospective study on lesion metric dynamics with a radiofrequency balloon assessed that fifteen seconds of radiofrequency delivery on posterior electrodes provides a good balance between safety, with no esophageal temperature rise, and efficacy with sufficient lesion metrics.

## Figures and Tables

**Table 1:** Lesion metric progression during 20 s of radiofrequency delivery. Impedance drop and temperature rise at the posterior electrodes from baseline to the plateau. The time to reach each percentile of the impedance and temperature plateau is also shown with the ratio of electrodes reaching those metrics’ values.

	Metrics	Baseline	90%	95%	Plateau
Impedance	Z (Ω)	102.4 (95.8–111.1)	86.6 (81.3.–92)	85.6 (80.4–91.1)	84.6 (79.3–90.2)
	ΔZ (Ω)	NA	16.6 (11–22.7)	17.5 (11.6–24)	18.4 (12.2–25.2)
	Time (s)	NA	10.2 (7.9–11.9)	11.8 (9.1–13.8)	13.9 (10.6–16)
	Electrodes’ distribution *	NA	99.6%	95.8%	75.6%
Temperature	T (°C)	27.1 (25.8–28.9)	34.2 (31.8–37.3)	36.1 (33.5–39.3)	38 (35.3–41.4)
	ΔT (°C)	NA	7.3 (3.7–10.8)	9.1 (5.3–12.8)	11.1 (7.1–14.9)
	Time (s)	NA	7.2 (3.9–9.6)	10.1 (6.4–13.1)	16.4 (12.6–18.4)
	Electrodes’ distribution **	NA	97.2 %	92.8%	47%

Continuous variables are shown as median (Q1–Q3) and categorical parameters as numbers and percentages. Z: Impedance, ΔZ: impedance drop at percentile of the plateau, s: seconds, * percentage of electrodes reaching 90–95–100% of the impedance drop plateau, T: temperature, ΔT: temperature rise, ** percentage of electrodes reaching 90–95–100% of the temperature rise plateau.

**Table 2:** Lesion metric progression comparison between ETR groups for a 20 s radiofrequency delivery. Comparison of impedance drop and temperature rise at each electrode from baseline to the plateau during a 20 s radiofrequency delivery between ETR groups. The time to reach each percentile of the impedance and temperature plateau is also shown with the ratio of electrodes reaching these metric values.

		Baseline			90%			95%			Plateau		
	Groups	ETR −	ETR +	*p* Value	ETR −	ETR +	*p* Value	ETR −	ETR +	*p* Value	ETR −	ETR +	*p* Value
Impedance	Z (Ω)	101.8 (95.5–110.5)	105.1 (98.3–115.5)	0.018	86.6 (81.4–92.2)	86.6 (79.6–90.6)	0.071	85.6 (80.5–91.4)	83.5 (78.4–89.3)	0.031	84.8 (79.4–90.6)	82.2 (77.1–87.5)	0.012
	ΔZ (20) (Ω)	NA	NA	NA	16 (10.1–22.1)	21.4 (15–28.5)	0.0001	16.9 (10.7–23.4)	22.6 (15.8–30)	0.0001	17.8 (11.2–24.6)	23.8 (16.7–31.7)	0.0001
	Time (s)	NA	NA	NA	10 (7.5–11.8)	10.4 (8.8–12)	0.048	11.5 (8.7–13.7)	12.5 (10.6–13.7)	0.009	13.6 (10–15,9)	14.7 (12.3–16.4)	0.005
Temperature	T (°C)	27.3 (26–29)	26.1 (25.1–28.1)	0.0001	34.2 (31.8–37.2)	26.1 (25.1–28.1)	0.001	36.1 (33.5–39.2)	37 (34.5–40.8)	0.002	38 (35.3–41.3)	39 (36.3–42.9)	0.002
	ΔT (20) (°C)	NA	NA	NA	7.1 (3.5–10.7)	9.1 (6.4–12.5)	0.0001	8.9 (5.1–12.7)	11.3 (8.1–14.7)	0.002	10.8 (6.9–14.8)	13.1 (9.9–16.9)	0.0001
	Time (s)	NA	NA	NA	7.1 (3.6–9.5)	8.6 (5.9–10.5)	0.0007	10 (6–13)	11.3 (8.7–14.2)	0.004	16.3 (12.3–18.4)	13.6 (10–15.9)	0.47

Continuous variables are shown as the median (Q1–Q3) and categorical parameters are shown as numbers and percentages. ETR-: Patients without an esophageal temperature rise above 42 °C; ETR+: patients with an esophageal temperature rise above 42 °C, Z: impedance, ΔZ (20): impedance drop at the 90–95–100% percentile of the plateau, s: seconds, T: temperature, ΔT (20): temperature rise at the 90–95–100% percentile of the plateau.

**Table 3:** Lesion metric value comparison between ETR groups after 15 s of radiofrequency delivery. Comparison of impedance drop and temperature rise at each electrode from baseline to 15 s of RF delivery between ETR groups. The time to reach each percentile of the plateau is shown. The ratio of electrodes reaching these metric values is also presented.

		Baseline			90%			95%			Plateau		
	Groups	ETR −	ETR +	*p* Value	ETR −	ETR +	*p* Value	ETR −	ETR +	*p* Value	ETR −	ETR +	*p* Value
Impedance	Z (15) (Ω)	86.6 (81.4–92.2)	85 (79.6–90.6)	0.02	85.7 (81.4–92.2)	83 (78.4–88.2)	0.01	85.2 (79.9–91.1)	81.5 (77.3–86.0)	0.001	86.6 (81.4–92.2)	85 (79.6–90.6)	0.02
	ΔZ (15) (Ω)	16 (10.1–22.1)	21.4 (15–28.5)	0.0001	16.5 (10.5–22.5)	22.2 (15.7–28.4)	0.0001	15.4 (9.7–21.5)	22.3 (16.1–27.6)	0.0001	16 (10.1–22.1)	21.4 (15–28.5)	0.0001
	Electrodes distribution *	99.5%	100%		92.3%	95.8%		76.4%	71.8%		99.5%	100%	
Temperature	T (15) (°C)	34.2 (31.7–31.7)	35.1 (32.7–32.7)	0.034	35.7 (33.4–33.4)	36.8 (34.3–34.3)	0.034	35.9 (34.5–34.5)	37.4 (35.7–35.7)	0.034	34.2 (31.7–31.7)	35.1 (32.7–32.7)	0.034
	ΔT at 15 s	7 (3.5–10.6)	9.1 (6.4–12.5)	0.001	8.5 (4.9–11.9)	10.9 (7.3–14.2)	0.001	7.6 (5–11.3)	11.2 (7–14.5)	0.003	7 (3.5–10.6)	9.1 (6.4–12.5)	0.001
	Electrodes distribution *	97.3%	100%		92.6%	93.6%		47.4%	46.1%		97.3%	100%	

Continuous variables are shown as the median (Q1–Q3) and categorical parameters are shown as numbers and percentages. ETR-: Patients without esophageal temperature rise above 42 °C; ETR+: patients with esophageal temperature rise above 42 °C, Z: impedance, ΔZ (15): impedance drop at each percentile of the plateau, s: seconds, T: temperature, ΔT (15): temperature rise at each percentile of the plateau. *: percentage of electrodes reaching 90–95–100% of the impedance drop or temperature rise plateau.

**Table 4:** Comparison of lesion metrics at the plateau in the ETR+ group between 15 and 20 s.

	At 15 s	At 20 s	*p* Value
ΔT_100%_ (°C)	11.2 (7–14.5)	13.1 (9.9–16.8)	0.11
ΔΩ_100%_ (Ω)	22.3 (16.1–27.6)	23.8 (16.7–31.7)	0.31

## Data Availability

The data underlying this article will be shared upon reasonable request to the corresponding authors.
